# Workplace and non-workplace loneliness: a comparative study on risk factors and impacts on absenteeism and mental health among employees in Spain

**DOI:** 10.1192/j.eurpsy.2025.394

**Published:** 2025-08-26

**Authors:** J. Domènech-Abella, C. Muntaner, J. Mundó, J. M. Haro

**Affiliations:** 1 Parc Sanitari Sant Joan de Déu, Sant Boi de Llobregat (Barcelona), Spain; 2 University of Toronto, Toronto, Canada; 3 Universitat de Barcelona; 4 Parc Sanitari Sant Joan de Déu, Barcelona, Spain

## Abstract

**Introduction:**

Loneliness can manifest in various aspects of life, including personal and professional contexts. Although most of studies on loneliness are focused on general loneliness without the specification on any context, the study of workplace loneliness has garnered increased attention in recent years and researchers explore how loneliness in professional settings affects labor satisfaction, productivity and mental health of workers. However, few studies have directly compared workplace and non-workplace loneliness, particularly in terms of their prevalence, agreement with each other, risk factors, and consequences

**Objectives:**

The aim of this study is to (1) evaluate prevalences and concordance between workplace and non-workplace loneliness, (2) compare sociodemographic risk factors between workplace and non-workplace loneliness, (3) compare working conditions-related risk factors between the two contexts of loneliness, and (4) compare their impact on absenteeism, depression, anxiety and substance use disorder.er.

**Methods:**

A representative sample of the employee residing in Spain (n=5400) was surveyed using computer-assisted web interviews (CAWI). Logistic regression models were constructed to compare the effects of risk factors for workplace and non-workplace loneliness (including sociodemographic factors, and factors related to working conditions), as well as the impact of workplace and non-workplace loneliness on absenteeism, and symptoms of depression, anxiety, and substance use disorder.

**Results:**

40,7% of active workers report experiencing workplace loneliness, while 42.0% report non-workplace loneliness. The level of concordance between both types of loneliness is low (k=0.36). Both types are more prevalent among younger workers and migrated people. Other sociodemographic risk factors (being female, non-married, and non-heterosexual) were significantly associated with non-workplace loneliness. Meanwhile, risk factors related to working conditions -particularly working under stress and labor precariousness- were associated with both types of loneliness, which showed an independent impact on absenteeism, depression, anxiety, and substance use disorder.

**Conclusions:**

Most of social determinants of workplace loneliness are rooted in the work environment, indicating that effective interventions should focus on addressing labor conditions and precariousness to improve both workplace and non-workplace loneliness and their impacts on absenteeism and mental health.

**Disclosure of Interest:**

None Declared

